# Molecular Prevalence of Human Astrovirus Among Children Under Five Years of Age With Gastroenteritis in Ilorin Metropolis, North‐Central Nigeria: A Cross‐Sectional Study

**DOI:** 10.1002/hsr2.73388

**Published:** 2026-10-13

**Authors:** Pius Omoruyi Omosigho, Toluwalope Serah Famuwagun, Eunice Korede Ogunleye, Adeleke Adewumi, Olalekan John Okesanya

**Affiliations:** ^1^ Department of Medical Laboratory Science Edo State University Uzairue Benin Nigeria; ^2^ Department of Medical Laboratory Science University of Ilorin Ilorin Kwara State Nigeria; ^3^ Department of Medical Microbiology and parasitology University of Ilorin Teaching Hospital Ilorin Nigeria; ^4^ Department of Laboratory Unit, University Health Centre Ladoke Akintola University of Technology Ogbomoso Nigeria; ^5^ Department of Research, Global Health Focus University of Rwanda Kigali Rwanda; ^6^ Department of Public Health, Faculty of Medicine and Health Sciences Hormuud University Mogadishu Somalia; ^7^ Department of Medical Laboratory Science, Neuropsychiatric Hospital Aro Neuropsychiatric Hospital Aro Abeokuta Nigeria

**Keywords:** astrovirus, child, gastroenteritis, HAstV, human astrovirus, preschool, prevalence

## Abstract

**Background and Aims:**

Human astrovirus (HAstV) is a recognized cause of viral gastroenteritis, however, data from north‐central Nigeria remain limited. This study aimed to determine the prevalence of classic HAstV RNA and explore associated factors among children under 5 years presenting with gastroenteritis in Ilorin, Nigeria.

**Methods:**

This hospital‐based cross‐sectional study was conducted from May 2025 to September 2025 among 200 children aged 13–59 months attending selected hospitals in Ilorin who presented consecutively. Stool samples were collected and analyzed using real‐time reverse transcription polymerase chain reaction for classic HAstV RNA. Data were analyzed using R software. Participant characteristics were summarized descriptively, prevalence estimates were calculated with exact 95% confidence intervals, and associations with astrovirus detection were evaluated using Fisher's exact test, the Wilcoxon rank‐sum test, and Firth penalized logistic regression.

**Results:**

Classic HAstV RNA was detected in 4 of 200 children under 5 years with acute gastroenteritis, giving an overall prevalence of 2.0% (4/200; 95% CI: 0.55%–5.04%). Prevalence was highest among children aged 49–60 months (2.94%), males (2.50%), rural residents (2.33%), users of borehole/well water (2.86%), and children attending daycare (4.0%). All astrovirus‐positive children presented with vomiting, moderate dehydration, resided in rural communities, had animal contact, used borehole/well water, and received oral rehydration solution. No other characteristics were significantly associated with HAstV. Feeding method was the only factor significantly associated with detection (*p* = 0.03), with the highest prevalence among exclusively breastfed children (33.3%; 2/6). However, wide confidence intervals around both the raw prevalence and the adjusted odds ratio (aOR = 0.01; 95% CI: 0.00–0.29) indicate this finding is exploratory rather than confirmed, warranting confirmation in larger studies.

**Conclusion:**

Astrovirus in this population is relatively low; however, it remains epidemiologically important, particularly as prevalence may vary with season. Continued surveillance and further molecular characterization are warranted.

## 1 Introduction

Astroviruses are non‐enveloped, positive‐sense single‐stranded ribonucleic acid (RNA) viruses and are well‐recognized causes of gastroenteritis in humans, particularly young children [1]. Although primarily associated with gastrointestinal illness, they have occasionally been linked to extra‐intestinal diseases [2]. The virus is primarily transmitted through the fecal–oral route [3], contaminated food and water, direct person‐to‐person transmission has also been reported [4]. In contrast to enveloped viruses, this virus is highly stable in the environment and can persist for extended periods in water, feces, and other materials [1]. Human astroviruses (HAstVs) belong to the genus Mamastrovirus, comprising classic strains (HAstV‐C, types 1–8) and the more recently described novel strains (HAstV‐MLB and HAstV‐VA/HMO), of which HAstV‐1 is the most commonly reported genotype globally [5, 6]. This virus has been identified as a significant cause of gastroenteritis, accounting for a pooled prevalence of 4.2% among children worldwide [7].

In children, astrovirus causes mild watery diarrhea that typically lasts 2–3 days and is often accompanied by vomiting, fever, loss of appetite, and abdominal pain [8]. They are typically self‐limiting, though co‐infections with other viruses, particularly rotavirus, sapovirus, and norovirus are common and have been linked to more severe symptoms, especially in low‐ and middle‐income country (LMIC) settings [9]. Based on the Global Burden of Disease (GBD) study in 2021, diarrheal disease caused an estimated 1.17 million deaths globally in 2021, with children under five remaining among the most affected groups [10]. To the best of our knowledge, this is the first RT‐PCR‐based assessment of classic human astrovirus among under‐five children with gastroenteritis in Ilorin metropolis. The only previous astrovirus study from Kwara State was conducted in Offa using ELISA, reporting a 31.3% prevalence of astrovirus among 300 children aged 5 years and below with gastroenteritis [11]. RT‐PCR‐based pediatric data within Nigeria are limited to Ogun and Osun States in the southwest, underscoring the need for the study in this region. Therefore, this study aimed to determine the prevalence of classic human astrovirus RNA among children under 5 years of age presenting with gastroenteritis in Ilorin Metropolis, Kwara State, and to explore demographic, environmental, behavioral, and household factors potentially associated with its detection using RT‐qPCR‐based molecular method.

## 2 Methods

### 2.1 Study Design and Setting

This was a hospital‐based cross‐sectional study conducted to determine the molecular prevalence of HAstV RNA and explore factors potentially associated with its detection among children under 5 years of age presenting with acute gastroenteritis in Ilorin Metropolis, Kwara State, North‐Central Nigeria. The study was conducted between May 2025 and September 2025 at the selected hospitals: Children Specialists' Hospital, Centre Igboro, Ilorin, and Kwara State University Teaching Hospital (KWASUTH). Acute gastroenteritis was defined as the occurrence of three or more loose or watery stools within a 24‐h period, with or without vomiting, lasting less than 14 days. Eligible participants were recruited consecutively during the study period until the sample size was attained. Data on children presenting with acute gastroenteritis who did not meet eligibility criteria, or who were eligible but declined participation, were not separately recorded. The minimum sample size was calculated using the single population proportion formula for cross‐sectional studies, assuming a prevalence of 19.4% reported among children with acute gastroenteritis in a Nigerian study using RT‐PCR [12], a confidence level of 95% (*Z* = 1.96), and a precision level of 5% (*d* = 0.05) to give a sample size of 240. However, due to limitations in available resources, a total of 200 eligible children were included in this study.

### 2.2 Eligibility Criteria

Children within the age of 0–59 months presenting clinically diagnosed acute gastroenteritis in either of the selected hospitals were considered eligible, provided their caregiver gave a written informed consent. However, no children younger than 13 months presented for enrollment during the study period; the analytic sample, therefore, comprised children aged 13–59 months. Children were excluded if they were 60 months old and above, immunocompromised, previously enrolled in the study, had chronic disease, or caregiver did not provide written informed consent.

### 2.3 Questionnaire Administration, Clinical Assessment, and Sample Collection

Information on socio‐demographic characteristics, clinical history, duration of illness, presenting symptoms, previous episodes of gastroenteritis, vaccination history, environmental exposures, household sanitation conditions, drinking‐water sources, hygiene practices, daycare attendance, and animal contact were obtained from participants' caregivers using a structured, interviewer‐administered questionnaire to minimize potential sources of bias. Height was measured to the nearest 0.1 cm. Five gram of fresh stool was collected into a sterile leak‐proof container, and each sample was given a unique identification number. Each stool sample was stored at −80°C after collection. Prior to analysis, samples were thawed, aliquoted into cryovials, and processed for RNA extraction.

### 2.4 Molecular Detection of Human Astrovirus

Stool samples were homogenized with phosphate buffered saline, centrifuged, and supernatant collected in a clean microtube for viral RNA extraction. Viral RNA was extracted using the ARGENE extraction reagents (bioMérieux, France) according to the manufacturer's instructions. The extracted RNA was subsequently subjected to real‐time reverse transcription polymerase chain reaction (RT‐qPCR) for the detection of classic human astrovirus (HAstV‐C) using the Diagnostic Kit for Sapporo virus type‐V/HumanAstrovirus Classic Type/Human Adenoviruses RNA (Real‐Time PCR Method) (Cat.# PD‐R‐1202B; Guangzhou Viuick Biotechnology Co. Ltd., Guangzhou, China) following manufacturer's protocol, with fluorescence detection in the VIC channel and CY5 used for internal control. Each run included a positive control, a negative control, and an internal control to validate the assay performance and reduce any potential sources of bias. A positive result was recorded when the threshold cycle (Ct) was less than or equal to 40 (Ct ≤ 40) in the VIC channel with a good S‐curve. A negative result was recorded when the Ct was greater than 40 (Ct > 40) or no Ct value in VIC. An invalid result was recorded when no VIC signal or the internal control (CY5) failed to amplify or was very late (Ct > 35).

### 2.5 Data Management and Statistical Analysis

Data obtained from questionnaires and laboratory analyses were entered into Microsoft Excel, cleaned, harmonized, and analyzed using R version 4.5.1 [13]. Prespecified analyses included the following: descriptive statistics were used to summarize participant characteristics, with continuous variables presented as mean ± standard deviation (SD) and categorical variables as frequencies and percentages. There was no missing data; complete data were available for all participants across all variables. The overall prevalence of human astrovirus RNA and category‐specific prevalence estimates were calculated and reported with exact 95% confidence intervals (95% CI) using the Clopper–Pearson method [14]. Participant characteristics were compared according to human astrovirus RNA detection status using the Wilcoxon rank‐sum test for continuous variables and Fisher's exact test for categorical variables, given the small number of positive cases; these exploratory bivariate analyses assessed associations between demographic, environmental, clinical, and behavioral factors and detection status. Detailed epidemiological profiling of all astrovirus‐positive cases was conducted. To further explore associations while addressing sparse outcome data, Firth penalized logistic regression [15] was performed using the logistf package, version 1.26.1, in R; this analysis was likewise exploratory, given the limited positive cases, rather than prespecified and confirmatory. Regression coefficients, adjusted odds ratios (AORs), 95% confidence intervals, and *p* values were reported. Model performance was assessed using likelihood ratio and Wald *χ* ^2^ statistics. Graphical analyses visualized overall and category‐specific prevalence, epidemiological profiles of positive cases, and the relative strength of associations identified through Fisher's exact tests. No subgroup analyses beyond those described above were conducted. All statistical tests were two‐sided and statistical significance was set a priori at *p* < 0.05. This report was prepared with reference to the SAMPL guidelines for statistical reporting.

### 2.6 Ethical Considerations

Ethical approval was obtained from the Kwara State Ministry of Health Ethical Research Committee (ERC) (Approval Number: ERC/MOH/2025/04/401). Written informed consent was obtained from parents or legal guardians of each participant. Caregivers were fully informed about the study procedures, objectives, benefits, risks, and their right to withdraw at any time without consequences. Anonymity of each participant was ensured before analysis.

## 3 Results

A total of 200 children aged 13–59 months presenting with acute gastroenteritis were enrolled in this study. Classic human astrovirus RNA was detected in four participants, yielding an overall prevalence of 2.0% (4/200; 95% CI: 0.55%–5.04%) in the study population (Figure 1). The characteristics of children with acute gastroenteritis stratified according to astrovirus RNA detection status are shown in Table 1, while the prevalence of astrovirus RNA according to demographic, environmental, and clinical characteristics is presented in Table 2. Figure 2 illustrates the distribution of astrovirus prevalence across selected participants and exposure characteristics, showing generally low prevalence estimates with wide and overlapping confidence intervals across most subgroups. No statistically significant association was observed between age group and astrovirus detection status (*p* = 0.66). Although astrovirus RNA were not identified among children aged 13–24 months, the prevalence was 1.59% (2/126) among children aged 25–48 months and 2.94% (2/68) among those aged 49–60 months. Of the four astrovirus‐positive children, two (50%) belonged to the 25–48 months age group and two (50%) were aged 49–60 months. Similarly, sex was not significantly associated with astrovirus detection (*p* > 0.99), although detection was slightly more common among males (2.50%; 2/80) than females (1.67%; 2/120), with positive cases equally distributed between both sexes. There was no significant difference in height characteristics between astrovirus‐positive and negative children (*p* = 0.70). Ethnicity was also not associated with detection status (*p* > 0.99); however, all positive cases occurred among Yoruba children. Likewise, no significant association was observed with local government area (*p*= 0.13), although positive cases were identified only in Asa and Ilorin West local government areas. Among the age categories, children aged 49–60 months exhibited the highest point prevalence (Figure 2). Residence type was not significantly associated with astrovirus detection (*p* > 0.99), but all astrovirus‐positive children resided in rural communities, resulting in a prevalence of 2.33% (4/172), whereas none were detected among urban residents. Environmental characteristics showed no statistically significant associations with astrovirus detection. All positive cases relied on borehole/well water sources, corresponding to a prevalence of 2.86% (4/140), while no positive cases were observed among children using borehole or tap water. Sanitation type was not significantly associated with detection (*p* = 0.70); however, the highest prevalence was observed among children using flush toilets (3.70%; 2/54), followed by pit latrines (1.54%; 2/130), while none were identified among children practicing open defecation or using other sanitation systems.

**Figure 1:**
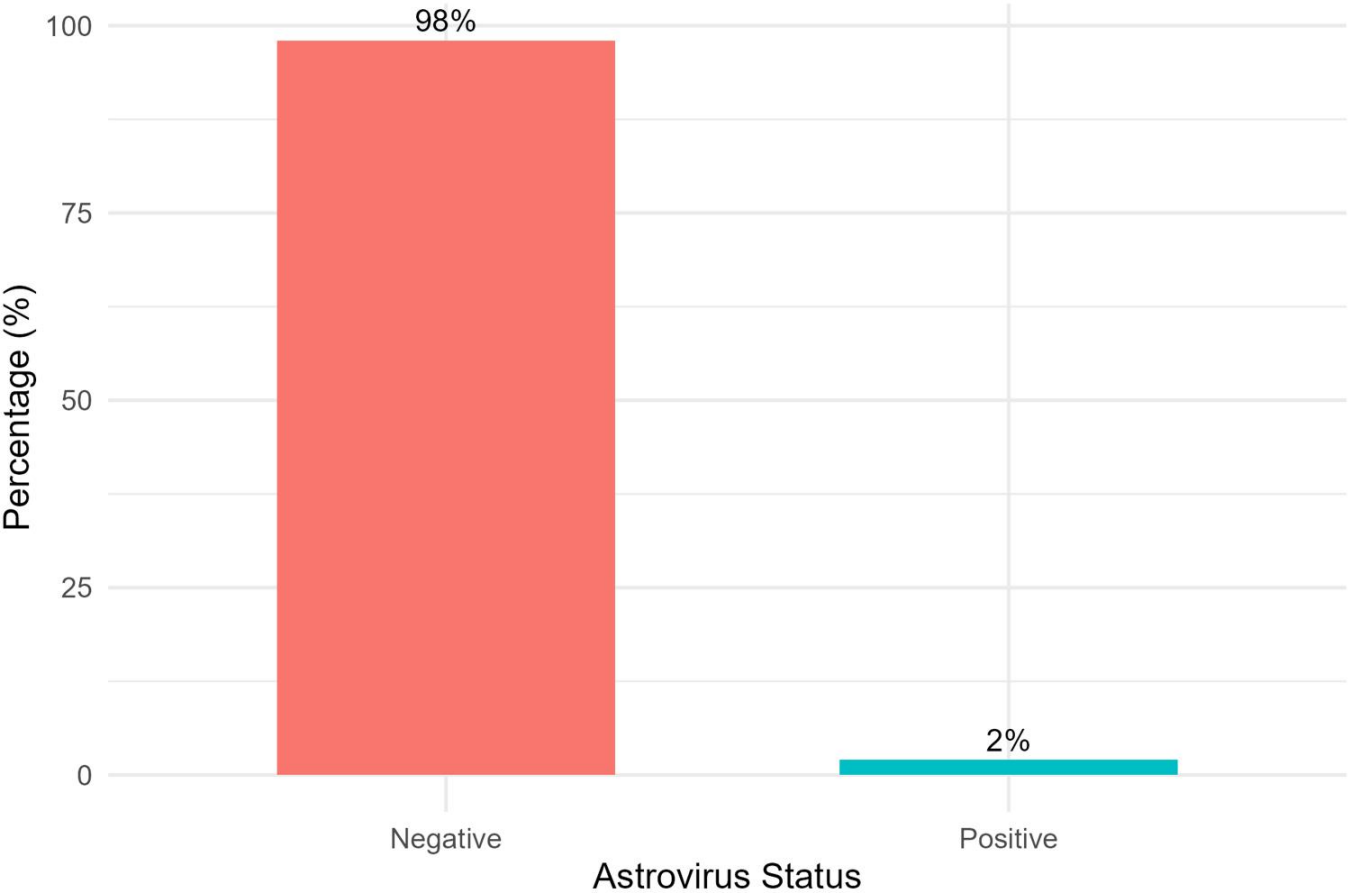
Prevalence of human astrovirus viral RNA among children with acute gastroenteritis in Ilorin Metropolis, North Central, Nigeria. Bar chart showing the percentage of astrovirus‐positive (2.0%, 4/200) and astrovirus‐negative (98.0% 196/200) stool samples among study participants (*N* = 200) as determined by RT‐PCR, giving an overall prevalence of 2.0%.

**Table 1:** Characteristics of children with acute gastroenteritis stratified by astrovirus RNA detection status.

Characteristic	Overall *N* = 200^a^	Negative *N* = 196^a^	Positive *N* = 4^a^	*p* value^b^
Age group^c^				0.66
13–24	6 (3.0%)	6 (3.1%)	0 (0%)	
25–48	126 (63%)	124 (63%)	2 (50%)	
49–60	68 (34%)	66 (34%)	2 (50%)	
Sex				> 0.99
Female	120 (60%)	118 (60%)	2 (50%)	
Male	80 (40%)	78 (40%)	2 (50%)	
Height (cm)	87.9 ± 4.0	87.9 ± 4.1	88.5 ± 1.9	0.70
Ethnicity				> 0.99
Hausa	8 (4.0%)	8 (4.1%)	0 (0%)	
Igbo	4 (2.0%)	4 (2.0%)	0 (0%)	
Yoruba	188 (94%)	184 (94%)	4 (100%)	
Local government area				0.13
Asa	32 (16%)	30 (15%)	2 (50%)	
Ilorin East	80 (40%)	80 (41%)	0 (0%)	
Ilorin South	24 (12%)	24 (12%)	0 (0%)	
Ilorin West	52 (26%)	50 (26%)	2 (50%)	
Others	12 (6.0%)	12 (6.1%)	0 (0%)	
Residence				> 0.99
Rural	172 (86%)	168 (86%)	4 (100%)	
Urban	28 (14%)	28 (14%)	0 (0%)	
Housing structure				0.12
Permanent	156 (78%)	154 (79%)	2 (50%)	
Temporary	20 (10%)	20 (10%)	0 (0%)	
Temporary/informal	24 (12%)	22 (11%)	2 (50%)	
Water source				0.75
Borehole	24 (12%)	24 (12%)	0 (0%)	
Borehole/well	140 (70%)	136 (69%)	4 (100%)	
Tap	36 (18%)	36 (18%)	0 (0%)	
Sanitation				0.70
Flush toilet	54 (27%)	52 (27%)	2 (50%)	
Open defecation	12 (6.0%)	12 (6.1%)	0 (0%)	
Others	4 (2.0%)	4 (2.0%)	0 (0%)	
Pit latrine	130 (65%)	128 (65%)	2 (50%)	
Caregiver education				0.19
None	4 (2.0%)	4 (2.0%)	0 (0%)	
Primary	73 (37%)	73 (37%)	0 (0%)	
Secondary	92 (46%)	90 (46%)	2 (50%)	
Tertiary	31 (16%)	29 (15%)	2 (50%)	
Caregiver occupation				0.43
Civil servant	40 (20%)	38 (19%)	2 (50%)	
Farmer	89 (45%)	87 (44%)	2 (50%)	
Other	11 (5.5%)	11 (5.6%)	0 (0%)	
Teacher	2 (1.0%)	2 (1.0%)	0 (0%)	
Trader	58 (29%)	58 (30%)	0 (0%)	
Diarrhea (days)				0.27
2	24 (12%)	24 (12%)	0 (0%)	
3	143 (72%)	141 (72%)	2 (50%)	
4	26 (13%)	24 (12%)	2 (50%)	
5	5 (2.5%)	5 (2.6%)	0 (0%)	
6	2 (1.0%)	2 (1.0%)	0 (0%)	
Symptoms				0.67
Abdominal pain	18 (9.0%)	18 (9.2%)	0 (0%)	
Diarrhea	32 (16%)	32 (16%)	0 (0%)	
Diarrhea, fever	2 (1.0%)	2 (1.0%)	0 (0%)	
Diarrhea, loss of appetite, sunken eyes	2 (1.0%)	2 (1.0%)	0 (0%)	
Fever	24 (12%)	24 (12%)	0 (0%)	
Sunken eyes	30 (15%)	30 (15%)	0 (0%)	
Vomiting	92 (46%)	88 (45%)	4 (100%)	
Dehydration				> 0.99
Mild	4 (2.0%)	4 (2.0%)	0 (0%)	
Moderate	186 (93%)	182 (93%)	4 (100%)	
None	4 (2.0%)	4 (2.0%)	0 (0%)	
Severe	6 (3.0%)	6 (3.1%)	0 (0%)	
Treatment				0.39
Antibiotics	44 (22%)	44 (22%)	0 (0%)	
Oral rehydration solution	94 (47%)	90 (46%)	4 (100%)	
Oral rehydration solution and antibiotics	2 (1.0%)	2 (1.0%)	0 (0%)	
ORS	48 (24%)	48 (24%)	0 (0%)	
ORS, Antib, IV	2 (1.0%)	2 (1.0%)	0 (0%)	
Others	10 (5.0%)	10 (5.1%)	0 (0%)	
Hospitalized				> 0.99
Yes	6 (3.0%)	6 (3.1%)	0 (0%)	
No	194 (97%)	190 (96.9%)	4 (100%)	
Rotavirus vaccine				0.24
Antibiotics	2 (1.0%)	2 (1.0%)	0 (0%)	
No	86 (43%)	86 (44%)	0 (0%)	
Not sure	20 (10%)	20 (10%)	0 (0%)	
Yes	92 (46%)	88 (45%)	4 (100%)	
Feeding method				0.03
Exclusive breastfeeding	6 (3.0%)	4 (2.0%)	2 (50%)	
Formula	2 (1.0%)	2 (1.0%)	0 (0%)	
Mixed feeding	180 (90%)	178 (91%)	2 (50%)	
Others	4 (2.0%)	4 (2.0%)	0 (0%)	
Solid food	6 (3.0%)	6 (3.1%)	0 (0%)	
Solid food/cooking food	2 (1.0%)	2 (1.0%)	0 (0%)	
Caregiver handwashing				> 0.99
Always	4 (2.0%)	4 (2.0%)	0 (0%)	
Others	4 (2.0%)	4 (2.0%)	0 (0%)	
Sometimes	192 (96%)	188 (96%)	4 (100%)	
Child handwashing				> 0.99
Always	4 (2.0%)	4 (2.0%)	0 (0%)	
Others	4 (2.0%)	4 (2.0%)	0 (0%)	
Rarely	2 (1.0%)	2 (1.0%)	0 (0%)	
Sometimes	190 (95%)	186 (95%)	4 (100%)	
Daycare (binary)	50 (25%)	48 (24%)	2 (50%)	0.26
Animal contact (binary)	190 (95%)	186 (95%)	4 (100%)	> 0.99

*Note: p* values are reported as follows: values < 0.001 as “*p* < 0.001”; values ≥ 0.001 and < 0.01 to the nearest thousandth; values ≥ 0.01 to the nearest hundredth; and values > 0.99, for example, 1.0000 reported as > 0.99.

^a^ *n* (%); mean ± SD.

^b^ Fisher's exact test; Wilcoxon rank sum test.

^c^ During the study period, no children younger than 13 months presented for enrollment; therefore, this age group is not represented.

**Table 2:** Prevalence of human astrovirus RNA according to demographic, environmental, and clinical characteristics of children with acute gastroenteritis.

Factor	Category	Number	Positive	Prevalence (%)
Age group	13–24 months	6	0	0.00
	25–48 months	126	2	1.59
	49–60 months	68	2	2.94
Sex	Female	120	2	1.67
	Male	80	2	2.50
Residence	Rural	172	4	2.33
	Urban	28	0	0.00
Water source	Borehole	24	0	0.00
	Borehole/well	140	4	2.86
	Tap	36	0	0.00
Sanitation	Flush toilet	54	2	3.70
	Open defecation	12	0	0.00
	Others	4	0	0.00
	Pit latrine	130	2	1.54
Rotavirus vaccine	Antibiotics	2	0	0.00
	No	86	0	0.00
	Not sure	20	0	0.00
	Yes	92	4	4.35
Feeding method	Exclusive breastfeeding	6	2	33.30
	Formula	2	0	0.00
	Mixed feeding	180	2	1.11
	Others	4	0	0.00
	Solid food	6	0	0.00
	Solid food/cooking food	2	0	0.00
Daycare/school attendance	No	150	2	1.33
	Yes	50	2	4.00
Animal contact	No	10	0	0.00
	Yes	190	4	2.11
Dehydration severity	Mild	4	0	0.00
	Moderate	186	4	2.15
	None	4	0	0.00
	Severe	6	0	0.00

**Figure 2:**
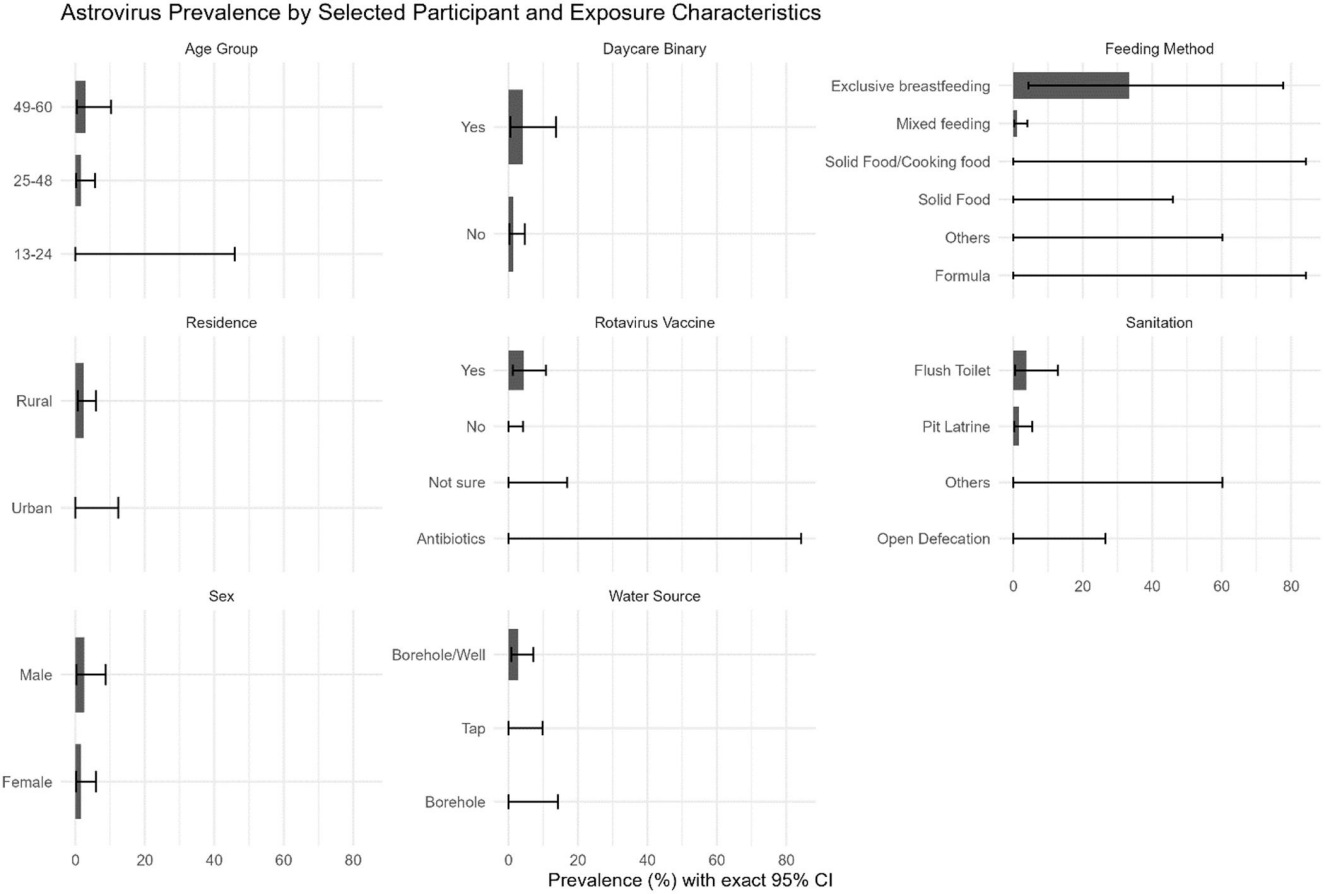
Distribution of astrovirus prevalence by selected risk factors. Estimated prevalence (%) of astrovirus across selected participant characteristics (water source, sex, age group, residence, feeding type, sanitation, rotavirus vaccine, and daycare binary) among study participants (*N* = 200). Error bars represent exact 95% confidence intervals calculated using the Clopper–Pearson method.

Caregiver educational level (*p* = 0.19) and occupation (*p* = 0.43) were not significantly associated with astrovirus detection. Positive cases were equally distributed among caregivers with secondary and tertiary education, and among those who were farmers and civil servants. Similarly, the duration of diarrhea (*p* = 0.27), symptom profile (*p* = 0.67), and dehydration status (*p* > 0.99) were not significantly associated with detection. Nevertheless, all infected children presented with vomiting and moderate dehydration, corresponding to a prevalence of 2.15% (4/186) among children with moderate dehydration. No positive case was observed among children with mild, severe, or no dehydration. Treatment received, hospitalization status, and rotavirus vaccination status were also not significantly associated with astrovirus detection (*p* = 0.39, (*p* > 0.99), and *p* = 0.24, respectively). Nonetheless, all four positive children had received oral rehydration solution and had reportedly been vaccinated against rotavirus, resulting in a prevalence of 4.35% (4/92) among vaccinated children. None of the hospitalized participants tested positive for astrovirus. Figure 2 further shows that prevalence estimates according to sex, daycare attendance, and rotavirus vaccination status were uniformly low with wide and extensively overlapping confidence intervals. Among all characteristics examined, the feeding method was the only factor significantly associated with astrovirus detection (*p* = 0.03). Half of the positive cases were exclusively breastfed, and the other half received mixed feeding. The prevalence of astrovirus was 33.3% (2/6) among exclusively breastfed children and 1.11% (2/180) among those receiving mixed feeding, whereas the virus was not detected among children receiving formula feeding, solid foods, or other feeding patterns. Consistent with this finding, exclusively breastfed children exhibited the highest point prevalence among all feeding categories shown in Figure 2, whereas prevalence estimates for the remaining feeding groups were substantially lower. No significant associations were found between caregiver handwashing practices (*p* > 0.99), child handwashing practices (*p* > 0.99), daycare attendance (*p* = 0.26), or animal contact (*p* > 0.99). However, astrovirus prevalence was higher among children attending daycare or school (4.0%; 2/50) compared with those not attending (1.33%; 2/150). Similarly, the viral RNA was detected only among children with animal exposure, corresponding to a prevalence of 2.11% (4/190), while it was not detected among children without animal contact.

Figure 3 illustrates the epidemiological and clinical profile of astrovirus‐positive cases. Each row represents a positive case for astrovirus, each column is a characteristic, and the color of each tile represents the value for that characteristic. All four cases had contact with animals, moderate dehydration, used borehole/well as water source, were not hospitalized, and resided in rural environment. They all experienced vomiting as a symptom and were treated with Oral Rehydration Solution. Figure 4 illustrates the exploratory ranking of factors associated with astrovirus detection based on Fisher's exact test. Results are presented as −log10‐transformed *p* values, with the reference line denoting the *p* = 0.05 threshold. The feeding method was the only characteristic significantly associated with astrovirus detection, indicated by the bar chart crossing the reference line. Rotavirus vaccination status, daycare attendance, age group, sanitation, and water source showed signals but did not reach the significance threshold. Sex, residence, dehydration, and animal contact showed no association with detection.

**Figure 3:**
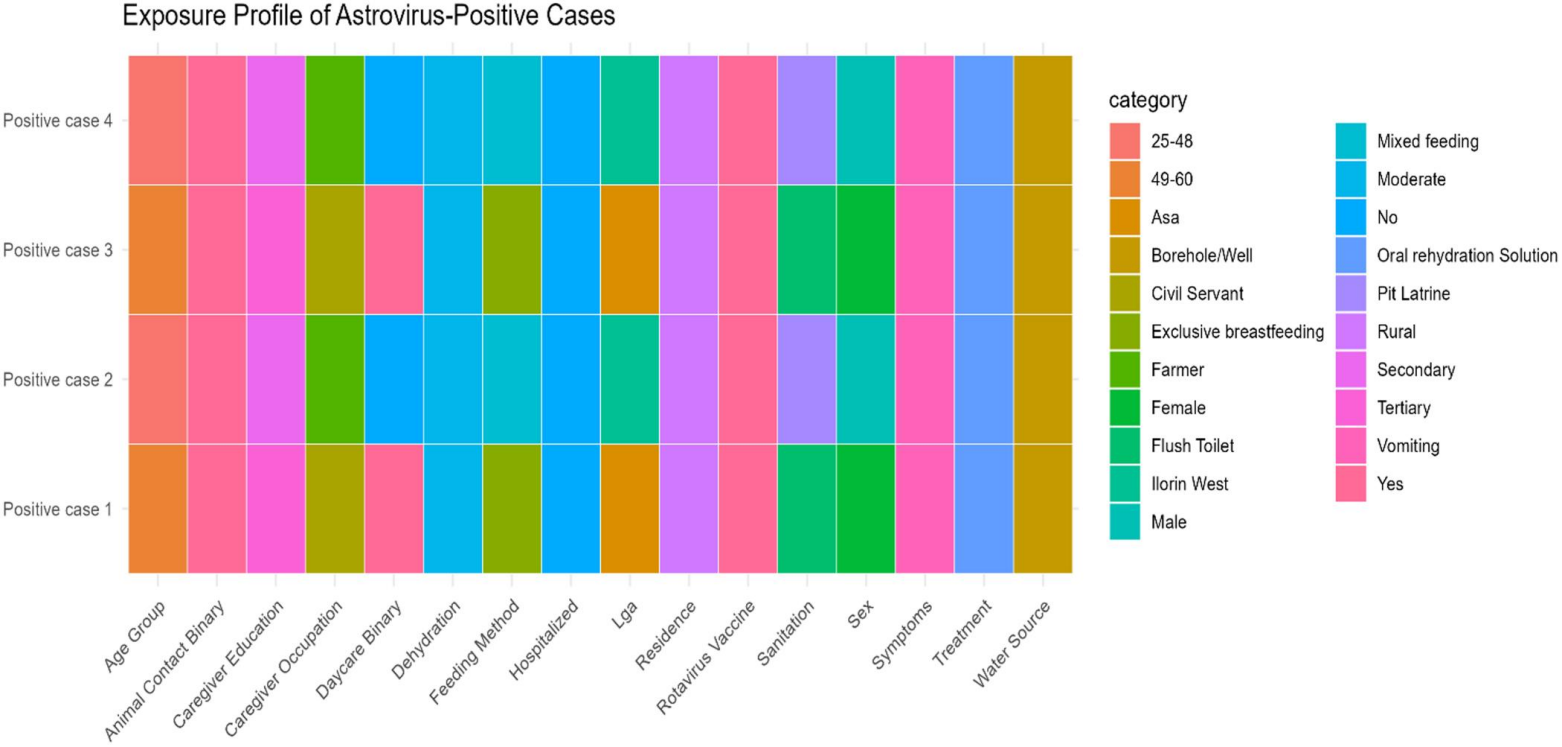
Epidemiological and clinical profile of astrovirus‐positive cases. Heatmap illustrating the distribution of demographic (age group, sex, residence, local government area, care giver education, care giver occupation) environmental (water source, sanitation, animal contact, daycare attendance), vaccination (rotavirus vaccination status), feeding (feeding method), and clinical characteristics (dehydration, treatment, symptoms) among the four astrovirus‐positive children identified in the study (rows: positive case 1–4). Each cell is color‐coded according to the specific category value recorded for that characteristic in each case, as indicated in the legend.

**Figure 4:**
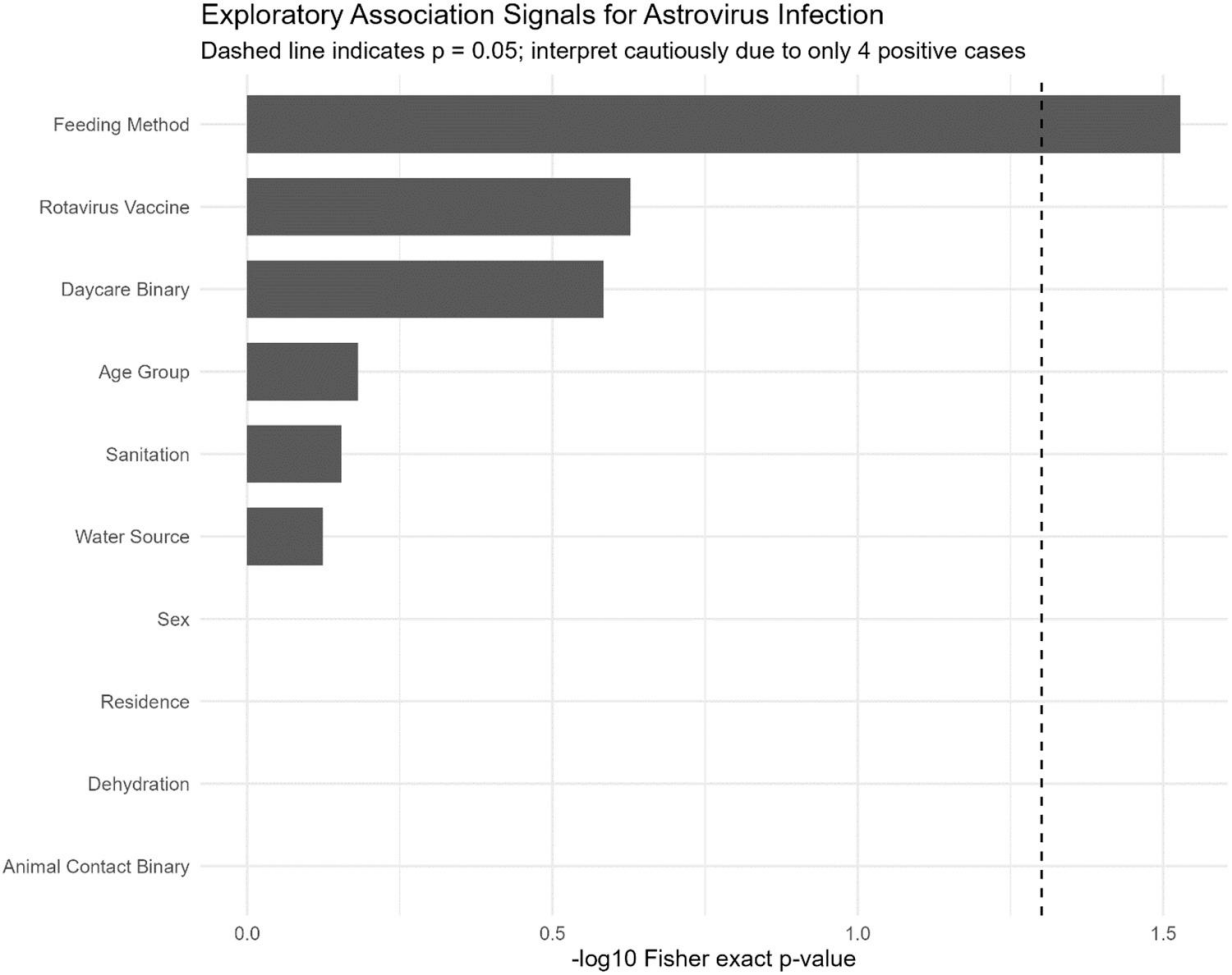
Exploratory ranking of factors associated with astrovirus detection based on Fisher's exact test. Legend: Bar chart ranking variables (feeding method, rotavirus vaccination status, daycare attendance, age group, sanitation, water source, sex, residence, dehydration, and animal contact) according to the strength of their association with astrovirus positivity among study participants (*N* = 200), using Fisher's exact test. Bar length represents −log10 (*p* value), such that longer bars indicate stronger associations (smaller *p* values). The dashed vertical line indicates the conventional significance threshold (*p* = 0.05). Results should be interpreted cautiously because of the small number of astrovirus‐positive cases (*n* = 4).

The exploratory bivariate analysis of factors associated with astrovirus detection using Fisher's exact test is presented in Table 3. The feeding method remained statistically significant in its association with astrovirus detection (*p* = 0.03). All other factors; age group (*p* = 0.66), sex (*p* > 0.99), residence (*p* > 0.99), water source (*p* = 0.75), sanitation (*p* = 0.70), rotavirus vaccine (*p* = 0.24), daycare (*p* = 0.26), animal contact (*p* > 0.99) and dehydration (*p* > 0.99) remained statistically insignificant. Table 4 represents the clinical and epidemiological profile of astrovirus‐positive cases (*n* = 4). Positive cases 1 and 3 were within the age group of 49–60 months, positive case 2 and 4 were within 25–48 months age group. Cases 1 and 3 were female, living in Asa local government area, used flush toilet, attended daycare, were exclusively breastfed, and had civil servant caregivers with tertiary education. Cases 2 and 4 were male, living in Ilorin West local government area, used pit latrine, did not attend daycare, had a mixed feeding diet with caregivers who were farmers with secondary school education. All four positive cases resided in a rural environment, used borehole/well as a water source, had moderated dehydration status with vomiting, and were treated with Oral Rehydration Solution and were not hospitalized. They had all been vaccinated with the rotavirus vaccine and exposed to animal contact.

**Table 3:** Exploratory bivariate analysis of factors associated with astrovirus detection using Fisher's exact test.

Variable	*p* value	Interpretation
Age group	0.66	No exploratory association
Sex	> 0.99	No exploratory association
Residence	> 0.99	No exploratory association
Water source	0.75	No exploratory association
Sanitation	0.70	No exploratory association
Rotavirus vaccine	0.24	No exploratory association
Feeding method	0.03	Exploratory association
Daycare	0.26	No exploratory association
Animal contact	> 0.99	No exploratory association
Dehydration	> 0.99	No exploratory association

*Note: p* values were obtained using Fisher's exact test because of the small number of astrovirus‐positive cases (*n* = 4) among study participants (*N* = 200). Results should be interpreted as exploratory and hypothesis‐generating rather than confirmatory. Variables with *p* < 0.05 were considered to show evidence of an exploratory association with astrovirus detection. Underlying category‐specific prevalence estimates are presented in Table 2 and Figure 2.

**Table 4:** Clinical and epidemiological profile of astrovirus‐positive cases (*n* = 4).

Characteristic	Positive case 1	Positive case 2	Positive case 3	Positive case 4
Age group	49–60	25–48	49–60	25–48
Sex	Female	Male	Female	Male
Residence	Rural	Rural	Rural	Rural
LGA	Asa	Ilorin West	Asa	Ilorin West
Water source	Borehole/well	Borehole/well	Borehole/well	Borehole/well
Sanitation	Flush toilet	Pit latrine	Flush toilet	Pit latrine
Caregiver education	Tertiary	Secondary	Tertiary	Secondary
Caregiver occupation	Civil servant	Farmer	Civil servant	Farmer
Rotavirus vaccine	Yes	Yes	Yes	Yes
Feeding method	Exclusive breastfeeding	Mixed feeding	Exclusive breastfeeding	Mixed feeding
Daycare attendance	Yes	No	Yes	No
Animal contact	Yes	Yes	Yes	Yes
Dehydration	Moderate	Moderate	Moderate	Moderate
Symptoms	Vomiting	Vomiting	Vomiting	Vomiting
Treatment	Oral rehydration solution	Oral rehydration solution	Oral rehydration solution	Oral rehydration solution
Hospitalized	No	No	No	No

The Firth penalized logistic regression analysis of potential factors associated with astrovirus detection among children under 5 years with gastroenteritis (*N *= 200) is presented in Table 5. Only the feeding method was significantly associated with astrovirus detection. Using exclusive breastfeeding as a reference, those on mixed feeding diet had significantly lower odds of astrovirus detection (aOR = 0.01; 95% CI: 0.00–0.29; *p* = 0.005). The remaining feeding categories, such as formula feeding (aOR = 0.15; *p* = 0.36), solid food (aOR = 0.13; *p* = 0.24), and the combined solid/cooking food group (aOR = 0.03; *p* = 0.10) showed reduced point estimates relative to exclusive breastfeeding but did not reach statistical significance. No other variable was significantly associated with its detection. Male sex was associated with higher point‐estimate odds of detection (aOR = 6.46; *p* = 0.14), while urban residence (aOR = 0.17; *p* = 0.46), daycare attendance (aOR = 0.70; *p* = 0.79), and animal contact (aOR = 1.13; *p* = 0.956) showed no significant association. Relative to the reference category, all levels of rotavirus vaccination status were non‐significant (*p* > 0.05). Table 6 shows the Firth penalized logistic regression model summary. The model estimated 12 parameters across the included predictors. The likelihood ratio test was not significant (*χ* ^2^_12 _= 16.53, *p* = 0.17). The Wald test had a significant result (*χ* ^2^_12 _= 53.51, *p* < 0.001).

**Table 5:** Firth penalized logistic regression analysis of factors associated with astrovirus detection among children under five years with gastroenteritis (*N* = 200).

Variable	*β* coefficient	SE	Adjusted OR (eβ)	95% CI for OR	*p* value
Sex					0.14
Female (ref)	—	—	1.00	—	
Male	1.866	1.102	6.46	0.56–936.39	
Residence					0.46
Rural (ref)	—	—	1.00	—	
Urban	−1.760	1.555	0.17	0.00–16.20	
Rotavirus vaccination status					—
Vaccination status/reference category	—	—	1.00	—	
No	−4.157	2.126	0.02	0.00–3.88	0.11
Not sure	−3.090	2.264	0.05	0.00–13.07	0.23
Yes	−2.438	1.901	0.09	0.00–17.32	0.28
Feeding method					—
Exclusive breastfeeding (ref)	—	—	1.00	—	
Formula	−1.903	2.061	0.15	0.00–7.27	0.36
Mixed feeding	−4.341	1.566	0.01	0.00–0.29	**0.005**
Others	−1.588	2.982	0.20	0.00–7094.95	0.68
Solid food	−2.051	1.731	0.13	0.00–3.16	0.24
Solid food/cooking food	−3.409	2.122	0.03	0.00–1.93	0.10
Daycare attendance					0.79
No (ref)	—	—	1.00	—	
Yes	−0.360	1.091	0.70	0.00–7.20	
Animal contact					0.96
No (ref)	—	—	1.00	—	
Yes	0.122	1.721	1.13	0.04–12586.38	

*Note: p* values are reported as follows: values < 0.001 as “*p* < 0.001”; values ≥ 0.001 and < 0.01 to the nearest thousandth; values ≥ 0.01 to the nearest hundredth; and values > 0.99, for example, 1.0000 reported as > 0.99. Bold *p* values indicate statistical significance at *p* < 0.05.

**Table 6:** Firth penalized logistic regression model summary.

Statistic	Value
Sample size (*N*)	200
Astrovirus‐positive cases	4
Likelihood ratio *χ* ^2^ (12 df)	16.53
Likelihood ratio *p* value	0.17
Wald *χ* ^2^ (12 df)	53.51
Wald *p* value	< 0.001
Regression method	Firth penalized logistic regression

*Note:* Model performance was assessed using likelihood ratio and Wald statistics. Firth penalized logistic regression was employed to reduce bias associated with the small number of astrovirus‐positive cases. *p* values are reported as follows: values < 0.001 as “*p* < 0.001”; values ≥ 0.001 and < 0.01 to the nearest thousandth; values ≥0.01 to the nearest hundredth; and values > 0.99, for example, 1.0000 reported as > 0.99.

## 4 Discussion

This study investigated the molecular prevalence of classic human astrovirus RNA in stool samples among children under 5 years of age presenting with gastroenteritis in Ilorin Metropolis, North‐Central Nigeria, and examined demographic, environmental, clinical, behavioral, and household factors potentially associated with astrovirus detection. Analyzed using RT‐PCR, an overall prevalence of 2.0% (4/200; 95% CI: 0.55%–5.04%) was observed. Although, the prevalence was relatively low, indicating that the virus is circulating among children with gastroenteritis in this setting. Compared to the prevalence reported in a similar population in Kwara state (31.3%) [11], the prevalence in this study is lower. Similarly, a study among Congolese children under five reported a higher astrovirus prevalence of 10.3% [16]. This could be attributed to the timing of sample collection. The Congolese study, which reported a higher prevalence, collected samples throughout an entire year (June 2012–2013) [16]. Similarly, a study conducted in Ogun State, Nigeria, which reported an even higher prevalence of 19.4%, also collected samples over an extended period (February 2015–April 2017) [12] compared to the present study, which was carried out over a period of 5 months (May 2025–September 2025).

In this study, all positive cases occurred among children aged 25–48 and 49–60 months, with none identified in the youngest group recruited (13–24 months; 0/6). A study in Ogun State, Nigeria, reported astrovirus detection across all age groups from 0 to 5 years, with a higher frequency in children aged 0–2 years [12]. The older age distribution observed here is therefore atypical, as the relatively immature immune system and lack of prior immunity in infants and younger children are generally thought to predispose them to enteric infections. This pattern most likely reflects the absence of participants aged 0–12 months and the limited number recruited in the youngest band, rather than a true age effect, and should not be interpreted as evidence that infants and younger children are at lower risk [17, 18].

The only significant characteristic associated with astrovirus detection was feeding method in both the exploratory bivariate analysis and the Firth penalized regression model, in which mixed feeding was associated with markedly lower odds of detection relative to exclusive breastfeeding. However, given the small number of positives in the population (4/200), this association should be considered as an exploratory. This association suggests the higher prevalence of astrovirus RNA among children who were exclusively breastfed (33.3%; 2/6) compared with those reported as mixed‐fed (1.11%; 2/180). However, the apparent effect is statistically fragile, as reflected in both the wide, overlapping confidence intervals around the raw prevalence estimates (Figure 2) and the extremely wide confidence interval around the adjusted odds ratio itself (aOR = 0.01; 95% CI: 0.00–0.29), a pattern consistent with model instability under sparse‐data conditions; the displacement of even a single case would substantially alter these estimates. The direction of association is also contrary to the well‐established protective role of breastfeeding in early childhood, which confers passive immunity against enteric infections [19]. It should be noted, however, that breastfeeding does not eliminate the influence of environmental exposures; such as rural residence and animal contact, which were common in this population, that may predispose children to enteric infection later in childhood [20, 21]. Taken together, these considerations suggest the observed association most likely reflects a small‐sample artifact rather than a true biological relationship, and it should be regarded as hypothesis‐generating rather than confirmatory.

Excluding the feeding method, none of the other variables (age group, sex, residence, water source, sanitation, daycare, animal contact, dehydration, and vaccination status) showed significant associations with astrovirus detection in this study. This finding should, however, be interpreted in the context of the small number of positive cases (*n* = 4), which left the analysis unable to detect associations of small or moderate magnitude; the absence of a significant association, therefore, cannot be taken as evidence that these factors are unrelated to astrovirus detection. This limitation is compounded by the homogeneity of the study population, as the majority of participants shared certain exposures; such as rural residence and animal contact, which further restricts the ability to detect associations with these variables. Consistent with this, a study in South Africa reported that many astrovirus‐infected children lived in contact with animals or livestock, were from low‐income households, and used pit latrines, although these associations did not reach statistical significance [22].

In this study, all four positive participants presented with vomiting and moderate dehydration, were managed with Oral Rehydration Solution, and none required hospitalization. This is consistent with the literature describing astrovirus as a cause of milder, self‐limiting gastroenteritis compared with other viral causes of acute gastroenteritis, such as rotavirus [23, 24]. All four astrovirus‐positive children had received rotavirus vaccination. This finding reflects the high vaccination coverage in the sample rather than a biological relationship; the rotavirus vaccine has no protective effect against astrovirus, and the association was not statistically significant on bivariate testing. This observation may, however, lend support to the hypothesis that as rotavirus vaccination reduces the burden of rotavirus‐associated gastroenteritis, non‐rotavirus enteric viruses such as astrovirus, norovirus, and sapovirus account for a relatively greater proportion of cases [25, 26, 27]. This underscores the importance of continued surveillance for non‐rotavirus enteric pathogens in the post‐vaccination era.

### 4.1 Strengths and Limitations

A key strength of this study was the use of RT‐qPCR for astrovirus detection, representing, to the best of our knowledge, the first molecular investigation of human astrovirus infection in Kwara State. The study also comprehensively assessed environmental, behavioral and household‐level factors potentially associated with astrovirus detection. Furthermore, the use of exact statistical tests and Firth penalized logistic regression was appropriate for addressing sparse data and the rare‐event nature of the outcome.

Several limitations should nevertheless be acknowledged. The calculated sample size of 240 participants was not achieved because of resource constraints, and only four astrovirus‐positive cases were identified. Together, these factors limited the statistical power to detect associations of small or moderate magnitude and reduced the precision and stability of subgroup and multivariable estimates, as reflected in the wide confidence intervals. Information on children who were assessed but did not satisfy the eligibility criteria or whose caregivers declined participation was not recorded separately. Therefore, a complete participant flow diagram could not be constructed, and the proportion of potentially eligible children who were ultimately enrolled could not be determined. The uneven distribution of participants across age groups and the exclusion of children younger than 13 months further limit the generalizability of the findings across the entire under‐five population, particularly given the substantial burden of diarrheal disease during infancy.

The cross‐sectional design precluded causal inference and limited the assessment of temporal relationships. In addition, the relatively short sampling period may not have adequately captured seasonal variations in astrovirus transmission. Because recruitment was restricted to two healthcare facilities in Ilorin Metropolis, the findings may not be representative of children in the wider community, other parts of Kwara State, or different healthcare settings. The RT‐qPCR assay targeted only classical human astroviruses (HAstV‐C); consequently, novel astrovirus lineages, including HAstV‐MLB and HAstV‐VA/HMO, were not detected. The reported prevalence may therefore underestimate the overall burden of human astrovirus infection in the study population. Furthermore, the absence of genotyping, sequencing, and phylogenetic analysis prevented the characterization of circulating genotypes, genetic diversity, and transmission relationships. Finally, participants were not screened for other enteric viruses, limiting the assessment of viral co‐infections and their possible contribution to the observed clinical manifestations.

### 4.2 Recommendations

Larger multicentre studies involving diverse populations are recommended to provide more robust estimates of the prevalence, burden, and determinants of astrovirus RNA detection among children in Kwara State and across Nigeria. Improvements in environmental sanitation, particularly sewage disposal systems, are necessary to reduce environmental contamination and interrupt fecal–oral transmission. These efforts should be complemented by strengthened health education programmes that promote regular handwashing, safe food preparation, and appropriate hygiene practices among caregivers. Future studies should also incorporate molecular characterization and genotyping of astrovirus strains, together with screening for viral co‐infections, to better understand the circulating variants and their contribution to disease severity. In addition, sustained surveillance of non‐rotavirus enteric viruses, including astrovirus, is warranted in the post‐rotavirus vaccination era to monitor changing epidemiological patterns and inform public health interventions.

## 5 Conclusion

This study identified a molecular prevalence of 2.0% for classical HAstV‐RNA among the children, with feeding method the only variable significantly associated with astrovirus detection, giving an exploratory and hypothesis‐generating association, further necessitating larger and more powered studies. Although the observed prevalence was low, the detection of HAstV remains epidemiologically relevant.

## Author Contributions

**Pius Omoruyi Omosigho:** conceptualization, supervision, writing – original draft, writing – review and editing, methodology. **Toluwalope Serah Famuwagun:** conceptualization, resources, methodology, writing – original draft, writing – review and editing, data curation. **Eunice Korede Ogunleye:** writing – review and editing, writing – original draft, conceptualization, methodology. **Adeleke Adewumi:** writing – original draft, writing – review and editing, data curation. **Olalekan John Okesanya:** writing – original draft, writing – review and editing, formal analysis.

## Funding

The authors have nothing to report.

## Ethics Statement

Ethical approval was obtained from the Ministry of Health Ethical Research Committee (ERC) ERC/MOH/2025/04/401 before study commencement. The study was conducted from May 2025 to September 2025.

## Consent

Written informed consent was obtained from the parents or legal guardians of all participating children prior to enrollment in the study. Participation was voluntary, and caregivers were informed about the study objectives, procedures, potential risks and benefits, confidentiality measures, and their right to withdraw at any stage without affecting the child's access to healthcare services.

## Conflicts of Interest

The authors declare no conflicts of interest.

## Transparency Statement

The lead author, Pius Omoruyi Omosigho affirms that this manuscript is an honest, accurate, and transparent account of the study being reported; that no important aspects of the study have been omitted; and that any discrepancies from the study as planned (and, if relevant, registered) have been explained.

## Data Availability

All datasets generated during this study are available from the corresponding author on reasonable request.

## References

[1] S. N. Roach and R. A. Langlois , “Intra‐ and Cross‐Species Transmission of Astroviruses,” Viruses 13 (2021): 1127. 10.3390/v13061127 PMC8230745 34208242

[2] A. B. Janowski , “Beyond the Gastrointestinal Tract: The Emerging and Diverse Tissue Tropisms of Astroviruses,” Viruses 13 (2021): 732. 10.3390/v13050732 PMC8145421 33922259

[3] V. Cortez , V. A. Meliopoulos , E. A. Karlsson , V. Hargest , C. Johnson , and S. Schultz‐Cherry , “Astrovirus Biology and Pathogenesis,” Annual Review of Virology 4 (2017): 327–348. 10.1146/annurev-virology-101416-041742 28715976

[4] S. Payne , “Family Astroviridae,” Viruses (2017), 125–128. ISBN 9780128031094.

[5] R. H. T. Nijhuis , I. A. Sidorov , P. K. Chung , et al., “PCR Assays for Detection of Human Astroviruses: In Silico Evaluation and Design, and In Vitro Application to Samples Collected From Patients in the Netherlands,” Journal of Clinical Virology 108 (2018): 83–89. 10.1016/j.jcv.2018.09.007 30266005

[6] M. Farahmand , P. Khales , Z. Salavatiha , et al., “Worldwide Prevalence and Genotype Distribution of Human Astrovirus in Gastroenteritis Patients: A Systematic Review and Meta‐Analysis,” Microbial Pathogenesis 181 (2023): 106209. 10.1016/j.micpath.2023.106209 37385570

[7] M. H. Razizadeh , K. Pourrostami , A. Kachooei , et al., “An Annoying Enteric Virus: A Systematic Review and Meta‐Analysis of Human Astroviruses and Gastrointestinal Complications in Children,” Reviews in Medical Virology 32 (2022): e2389. 10.1002/rmv.2389 36030520

[8] M. Mehdipour Dalivand , M. Ali , and R. Yee , “Prevalence and Clinical Manifestation of Astrovirus Gastroenteritis in Adults: A Seven‐Year Study in Washington D.C., USA,” Viruses 17 (2025): 730. 10.3390/v17050730 PMC12115731 40431741

[9] R. J. Rubinstein , L. Gutiérrez , C. Toval‐Ruíz , et al., “Epidemiology of Pediatric Astrovirus Gastroenteritis in a Nicaraguan Birth Cohort,” Open Forum Infectious Diseases 11 (2024): ofae465. 10.1093/ofid/ofae465 PMC11378399 39247803

[10] H. H. Kyu , A. Vongpradith , R. M. V. Dominguez , et al., “Global, Regional, and National Age‐Sex‐Specific Burden of Diarrhoeal Diseases, Their Risk Factors, and Aetiologies, 1990‐2021, for 204 Countries and Territories: A Systematic Analysis for the Global Burden of Disease Study 2021,” Lancet Infectious Diseases 25 (2025): 519–536. 10.1016/S1473-3099(24)00691-1 PMC12018300 39708822

[11] P. Omosigho , O. Izevbuwa , E. Rotimi , J. O. Okesanya , and M. Othoigbe , “Prevalence and Risk Factors of Astrovirus Gastroenteritis in Children in Offa, Kwara State, North Central Nigeria,” Microbes and Infectious Diseases 3 (2022): 596–605.

[12] K. O. Arowolo , C. I. Ayolabi , I. A. Adeleye , B. Lapinski , J. S. Santos , and S. M. Raboni , “Molecular Epidemiology of Astrovirus in Children With Gastroenteritis in Southwestern Nigeria,” Archives of Virology 165 (2020): 2461–2469. 10.1007/s00705-020-04741-0 PMC7414288 32770482

[13] R Core Team , R: A Language and Environment for Statistical Computing (R Foundation for Statistical Computing, 2025).

[14] C. J. Clopper and E. S. Pearson , “The Use of Confidence or Fiducial Limits Illustrated in the Case of the Binomial,” Biometrika 26 (1934): 404–413.

[15] D. Firth , “Bias Reduction of Maximum Likelihood Estimates,” Biometrika 80 (1993): 27–38.

[16] B. Nguekeng Tsague , V. Mikounou Louya , F. Ntoumi , et al., “Occurrence of Human Astrovirus Associated With Gastroenteritis Among Congolese Children in Brazzaville, Republic of Congo,” International Journal of Infectious Diseases 95 (2020): 142–147. 10.1016/j.ijid.2020.02.056 32194237

[17] D. L. Vu , A. Bosch , R. Pintó , and S. Guix , “Epidemiology of Classic and Novel Human Astrovirus: Gastroenteritis and Beyond,” Viruses 9 (2017): 33. 10.3390/v9020033 PMC5332952 28218712

[18] L. A. Moser and S. Schultz‐Cherry , “Pathogenesis of Astrovirus Infection,” Viral Immunology 18 (2005): 4–10. 10.1089/vim.2005.18.4 15802949

[19] A. A. Alotiby , “The Role of Breastfeeding as a Protective Factor Against the Development of the Immune‐Mediated Diseases: A Systematic Review,” Frontiers in Pediatrics 11 (2023): 1086999. 10.3389/fped.2023.1086999 PMC9981158 36873649

[20] B. Bertin , B. Foligne , D. Ley , et al., “An Overview of the Influence of Breastfeeding on the Development of Inflammatory Bowel Disease,” Nutrients 15 (2023): 5103. 10.3390/nu15245103 PMC10745409 38140362

[21] A. Purkiewicz , K. J. Regin , W. Mumtaz , and R. Pietrzak‐Fiećko , “Breastfeeding: The Multifaceted Impact on Child Development and Maternal Well‐Being,” Nutrients 17 (2025): 1326. 10.3390/nu17081326 PMC12030032 40284191

[22] R. Khumela , J. P. Kabue , A. N. Traore , and N. Potgieter , “Human Astrovirus in Symptomatic and Asymptomatic Children: A Cross‐Sectional Study on Hospitalized and Outpatients From Rural Communities of South Africa Between 2017–2021,” Pathogens 10 (2021): 1398. 10.3390/pathogens10111398 PMC8618259 34832554

[23] R. Saha , M. Lo , P. De , et al., “Epidemiology of Viral Gastroenteritis in Children and Genetic Diversity of Rotavirus Strains in Kolkata, West Bengal After Introduction of Rotavirus Vaccine,” Vaccine 45 (2025): 126637. 10.1016/j.vaccine.2024.126637 39731817

[24] X. L. Pang and T. Vesikari , “Human Astrovirus‐Associated Gastroenteritis in Children Under 2 Years of Age Followed Prospectively During a Rotavirus Vaccine Trial,” Acta Paediatrica 88 (1999): 532–536. 10.1080/08035259950169549 10426176

[25] P. O. Omosigho , V. W. Somtochukwu , and O. J. Okesanya , “Prevalence and Risk Factors of Norovirus Gastroenteritis in Children Under Five Years in Okada, Edo State, Nigeria,” Journal of Global Health Science 7 (2025): e10.

[26] P. O. Omosigho , U. V. Osayekewmen , G. P. Oghogho , O. J. Okesanya , J. M. Oladejo , and U. P. Osarodion , “Prevalence of Rotavirus Antigen in Children With Gastroenteritis in Auchi Etsako West Local Government Area, Edo State, Nigeria,” Le infezioni in Medicina 32 (2024): 69–75. 10.53854/liim-3201-9 PMC10917567 38456028

[27] C. L. D. Mboungou , C. C. M. Mapanguy , D. D. Anh , et al., “Beyond Rotavirus: Persistence of Norovirus, Adenovirus, and Astrovirus in Paediatric Gastroenteritis in the Republic of Congo After Vaccine Introduction,” Journal of Medical Virology 98 (2026): e70896. 10.1002/jmv.70896 PMC13071788 41969129

